# MiSDEED: a synthetic data engine for microbiome study power analysis and study design

**DOI:** 10.1093/bioadv/vbac043

**Published:** 2022-06-16

**Authors:** Philippe Chlenski, Melody Hsu, Itsik Pe’er

**Affiliations:** Department of Computer Science, Columbia University, New York, NY 10027, USA; Department of Computer Science, Columbia University, New York, NY 10027, USA; Department of Computer Science, Columbia University, New York, NY 10027, USA; Department of Systems Biology, Columbia University, New York, NY 10027, USA; Data Science Institute, Columbia University, New York, NY 10027, USA

## Abstract

**Summary:**

MiSDEED (*Mi*crobial *S*ynthetic *D*ata *E*ngine for *E*xperimental *D*esign) is a command-line tool for generating synthetic longitudinal multinode data from simulated microbial environments. It generates relative-abundance timecourses under perturbations for an arbitrary number of time points, samples, locations and data types. All simulation parameters are exposed to the user to facilitate rapid power analysis and aid in study design. Users who want additional flexibility may also use MiSDEED as a Python package.

**Availability and implementation:**

MiSDEED is written in Python and is freely available at https://github.com/pchlenski/misdeed.

## 1 Introduction

The behavior of the microbiome, partially elucidated by improvements in genome sequencing and data analysis, is generating considerable research interest. For instance, the Human Microbiome Project (Turnbaugh *et al.*, 2007) endeavors to collect data on a mass scale to investigate the role of the microbiome in the context of human health and disease. However, despite improvements in sequencing, sample collection itself still incurs significant overhead and many niches remain understudied. Furthermore, microbial relative-abundance data, the most typical form of data collected in such studies, have a number of properties that make classical statistical analysis challenging: it is longitudinal, compositional, noisy and stochastic (Bodein *et al.*, 2019). Thus, investigators who wish to study specific ecosystems or develop new tools for inference on such datasets must commit to the costly process of gathering real data at scale, generate synthetic data from scratch or submit to potentially inappropriate assumptions of conventional power analysis.

The genetic power calculator (Purcell *et al.*, 2003) streamlined research in statistical genetics by facilitating closed-form power analysis of hypothetical studies. Similarly, several tools help design microbiome studies: Web-GLV (Kuntal *et al.*, 2019) enables researchers to visualize the dynamics of microbial systems using assumed ecological parameters, and Mattiello *et al.* (2016) provide a power calculator for case-control studies on microbial ecosystems near equilibrium. The steady-state assumptions underlying the closed-form estimation of statistical power here can be inappropriate in trajectory-dependent contexts such as dynamical systems with chaotic responses to noise or studies examining the dynamics of a system’s transition between steady states. Moreover, for data-dependent applications such as machine learning model development, closed-form estimates of statistical power are unhelpful, whereas direct access to the actual underlying simulation is useful. The generalized Lotka-Volterra (gLV) modeling assumptions underlying the steady-state characterization are already used to generate synthetic data when designing inference methods for longitudinal microbial relative-abundance data (Joseph *et al.*, 2020a).

Here, we present MiSDEED: the *Mi*crobial *S*ynthetic *D*ata *E*ngine for *E*xperimental *D*esign, a flexible tool for generating synthetic longitudinal data from dynamic simulated ecosystems. Synthetic data generated by MiSDEED can be used in study design to simulate the analysis of real data collected under varying regimes, or in machine learning for model design and transfer learning.

## 2 Overview

### 2.1 Generative model

**Fig. 1.**
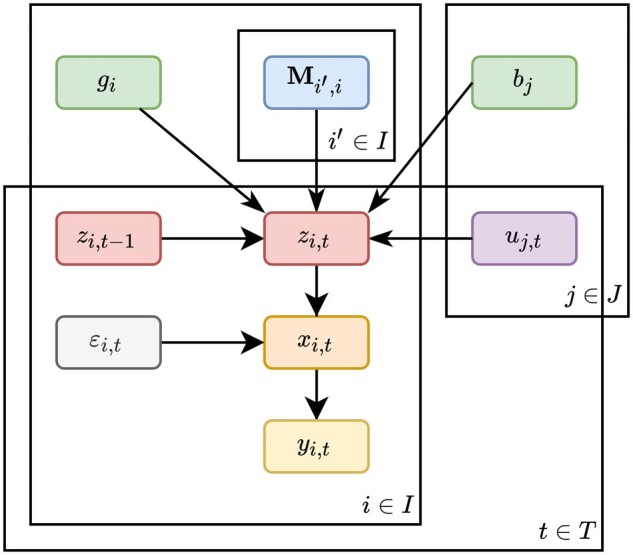
The graphical model underlying MiSDEED’s synthetic data engine. The abundance vector for node *i* at time *t*, $z_{i,t}$ is determined by the growth rates *g_i_*. The *y_t_* vectors are sampled from a multinomial distribution parameterized by the total number of reads and the probability vector *x_t_*

MiSDEED’s synthetic data generator (drawn in Fig. 1) samples reads from probability distributions governed by gLV dynamics over a discrete set of time points *T*. Each generator has a set *I* of nodes, which may represent different data types (e.g. metagenomics and metabolomics measurements of the same system) or two interacting ecosystems with the same data type.

**Fig. 2.**
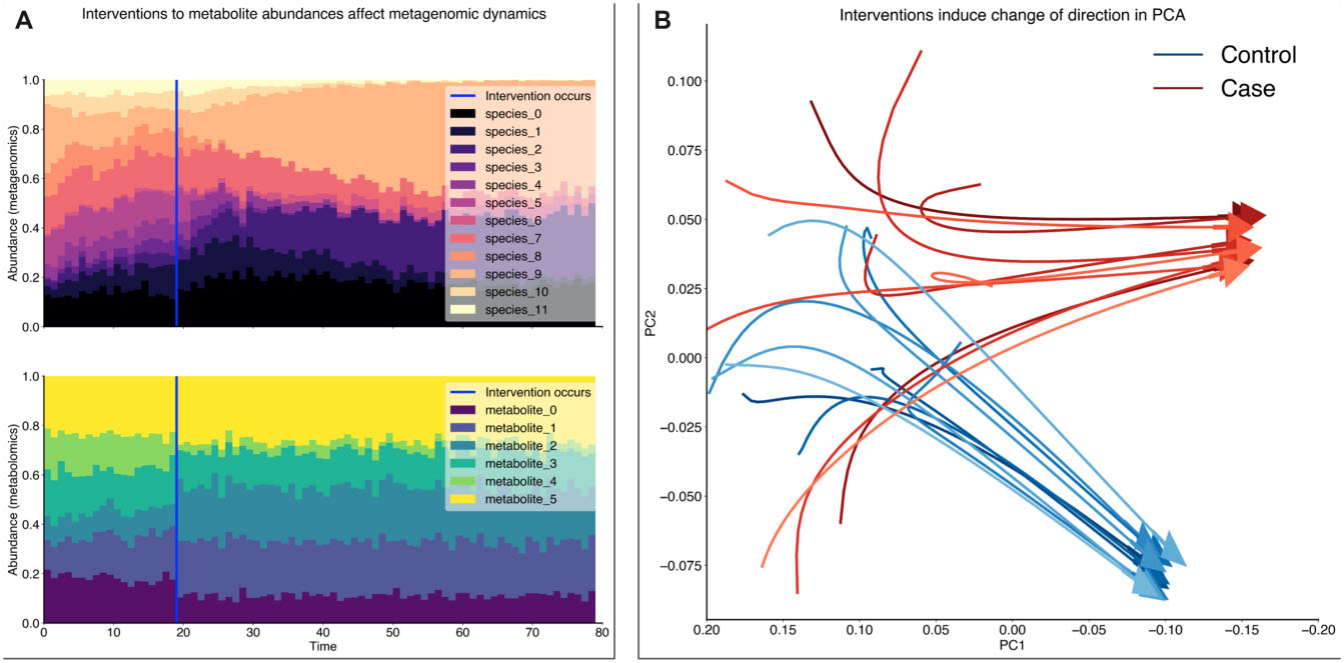
(**A**) Simulated metagenomic (top) and metabolomic (bottom) relative-abundance timecourses with an intervention at *t* = 20 (blue line). This intervention affects metabolite abundances directly and propagates into the metagenomics node gradually via metabolomics–metagenomics interactions. (**B**) Twenty noiseless PCA-transformed case and control metagenomic trajectories show how interventions induce convergence to distinct fixed points

Each node i∈I is initialized with a fixed dimensionality *d_i_*, a vector of growth rates $\vec{g_{i}}$, and an initial abundance vector $z_{i,0}$. A generator also has up to $|I{|}^{2}$ pairwise directed interactions between nodes. An interaction $M_{i,i\prime }$ between some nodes *i* and i′ is a matrix of dimension $d_{i}\times d_{i\prime }$. The matrix $M_{i,i\prime }$ describes the elementwise effects of each element in node *i* on each element in node i′. Finally, the generator has a set *J* of interventions which may be applied to any node i∈I such that each intervention *j* has a vector *u_j_* of intervention magnitudes and another vector *b_j_* of responses to the intervention. If intervention *j* is applied to node *i*, then *u_j_* should have |T| dimensions and *b_j_* should have *d_i_* dimensions.

Once generator parameters have been set, synthetic data can be produced in one of three ways: as a single timecourse, as multiple timecourses from varying initial conditions or as multiple timecourses following a case-control split. In each case, the generator numerically solves the following equations with a biological noise term *ε*:

(1) $$\begin{array}{c} \varepsilon ∼N\left(\vec{0},\;\sigma I\right)\\ \frac{dz_{i,t}}{dt}=z_{i,t-1}⊙(g_{i}+\sum_{i\prime \in I}M_{i,i\prime }z_{i\prime ,t}+\sum_{j\in J}u_{j,t}b_{j}+\varepsilon ). \end{array}$$

Each timecourse contains three derived matrices of synthetic data: **Z** (latent absolute abundances), **X** (latent relative abundances/probabilities) and **Y** (observed relative abundances sampled from **X**). To simulate read sampling, at each time point *t* a fixed number of reads *R* is drawn according to a multinomial distribution parameterized by the relative abundances *X_t_*, i.e. the *t*-th row of **X**:

(2) $$Y_{t}∼\mathrm{Multinomial}(R,X_{t}).$$

This generative process is outlined graphically in Figure 1.

Each gLV parameter (the growth rates *g_i_*, initial abundances $z_{i,0}$ and interaction matrices $M_{i,i\prime }$) can be specified by the user. Guidelines for convenient inference of gLV parameters can be found in the ‘parameter inference’ section. In practice, users may not have good estimates of gLV parameters. In these circumstances, the generator defines the gLV parameters according to the following distributions:

(3) $$\begin{array}{c} g_{i}∼U(-1,1)\\ z_{i,0}∼\mathrm{Logn}(0,1) \end{array}.$$

Interaction matrices are generated using the method described in Allesina and Tang (2012): first a square *D* × *D* matrix **M** is constructed, where

(4) $$D=\sum_{i\in I}d_{i}$$

is the sum of all of the node dimensions in the generator. Then, off-diagonal symmetric entries of $M_{i,j}$ and $M_{j,i}$ where i≠j are populated by pairs drawn from the following multivariate normal distribution:

(5) $$\langle M_{i,j},\;M_{j,i}\rangle ∼N\left(\langle 0,\;0\rangle ,\;\left[\begin{array}{cc} 1 & \rho \\ \rho & 1 \end{array}\right]\right).$$

The entries of **M** are subsequently sparsified according to the connectivity parameter *C* such that each entry is turned to 0 with probability 1−C:

(6) $$\begin{array}{c} I_{ij}∼\mathrm{Bernoulli}(C)\\ M_{ij}=M_{ij}*I_{ij} \end{array}.$$

The diagonal entries $M_{i,i}$ of **M** are set to *d*, the self-interaction penalty. Finally, the matrix **M** is divided up into node–node interaction matrices according to the dimensions of each node: the square submatrices of size $d_{i}\times d_{i}$ on the diagonals become self-interactions, whereas off-diagonal submatrices become cross-node interactions. The parameters for generating the interactions can be set by the user, as noted in Table 1.

**Table 1.** Variable parameters in MiSDEED

Category	Parameters
Generator	Number of time points, number of nodes, node names, node dimensions, time to first sample
Random interaction matrices	*C* (connectivity), *d* (negative self-interaction size), σ (multivariate normal variance), ρ (multivariate normal correlation)
Custom gLV parameters	Interaction matrices, growth rates, initial abundances, interventions and intervention responses
Synthetic data generation	Biological noise variance, number of reads, time step size, downsampling rate
Multiple samples	Number of individuals, probability of 0-valued initial abundances
Case-control	Case-control ratio, intervention node, intervention effect size

### 2.2 Generalized generative model

The previous section described the generation of a single timecourse, which would correspond to a single community. Users may wish to generate multiple timecourses at once, for instance simulating an entire community of patients at once. MiSDEED allows partial or complete sharing of gLV parameters and sampling parameters across individuals. By default, all gLV parameters are kept constant with the exception of initial abundances $z_{i,0}$, which are randomly drawn from the same distribution for each individual.

MiSDEED also provides rudimentary case-control functionality: interventions are modeled as an always-on intervention with a random response vector parameterized by the effect size *s*:

(7) $$\begin{array}{c} u_{C}=\vec{1}\\ b_{c}∼U(-s/2,\;s/2) \end{array}.$$

### 2.3 Usage

MiSDEED is designed to be used as a standalone command-line tool. The MiSDEED repository also contains the Python package underlying MiSDEED, a handful of utility scripts to support data visualization and learning gLV parameters and a set of Jupyter notebooks showing common uses of the MiSDEED Python package. MiSDEED can produce, save and plot large amounts of synthetic data with varying initial conditions and model assumptions. To support power analysis, many variables can freely be changed by the user. These are listed in Table 1.

### 2.4 Visualization

The MiSDEED command-line tool and Python package contain two functions for data visualization. One function allows the visualization of individual timecourses as stacked bar plots, as seen in Figure 2, Subplot A. This is one of the most typical data visualization used for microbial relative-abundance data, and it allows inspection of changes in relative abundance over time or in response to interventions. When stacked bar plots are generated for multiple systems at once, they can provide some intuition into the convergence time and existence of stable attractive states for a particular system of equations. A second function allows the visualization of many trajectories at once by projecting *d_i_*-dimensional trajectories down to their top two principal components, similar to the plot in Figure 2, Subplot B. Principal component analysis (PCA) visualizations facilitate easy inspection of trajectory convergence and change of states under interventions. For example, in Figure 2, Subplot B, it becomes evident that the case and control trajectories diverge in PCA space, corroborating the intuition that their differences should be detectable by an appropriate choice of statistical test and experimental conditions.

### 2.5 Power analysis example

As an example use case, one may use a community matrix and growth rates learned from a pilot dataset and initialize ‘metagenomics’ and ‘metabolomics’ nodes such that the latter has no intrinsic growth rates or self-interactions, but interactions with the ‘metagenomics’ node according to some *a priori* assumptions. Perturbing metabolite abundances directly, the user may investigate how many patients must be enrolled in order to distinguish reliably between samples with and without this perturbation applied.

In our example, we demonstrate that for a fixed number of study participants, increasing the read depth has a marked effect on the probability of observing a statistically significant difference between samples. For a study with 30 participants, it is sufficient to use a read depth of 300 to distinguish between case and control samples with high probability.

A Jupyter notebook demonstrating a detailed approach to power analysis using MiSDEED generators is included in the MiSDEED Github repository.

### 2.6 Parameter inference

Many users may find themselves in the situation of having some pilot dataset they wish to use as a basis for their simulations, but no existing gLV parameters. To facilitate the use of MiSDEED in this common situation, an implementation of the method for gLV trajectory inference given in Stein *et al.* (2013) is provided as part of the MiSDEED Python package. Using the notation used earlier in this paper, the Stein *et al.* (2013) method consists of computing the matrix

(8) $$(M_{i,i},g_{i},b_{i})=FY^{T}{\left(YY^{T}+D_{\lambda }\right)}^{-1},$$

where **F** is the matrix of time-scaled changes in *z_t_* between successive time points and **Y** is a row-by-row concatenation of the abundances *z_t_*, a vector $\vec{1}$ of 1s and all time-varying intervention indicator vectors *u_i_*.

It is worth noting that this method is sensitive to noise, demands a relatively large sample size in order to be accurate and presumes that absolute abundances are observed. Users who wish to do inference on relative-abundance trajectories should consider looking into the compositional Lotka-Volterra method laid out in Joseph *et al.* (2020b).

### 2.7 Data realism

Researchers considering the use of MiSDEED for power analysis may naturally be curious about benchmarking the realism of the MiSDEED-synthesized data according to some metrics of interest or altering the generative model underlying MiSDEED to more closely match an alternative set of assumptions about the way that their data should look.

We provide a Jupyter notebook demonstrating how all probability distributions can be overridden in the MiSDEED package, as well as a general outline of how data realism can be compared across synthetic and empirical datasets. The empirical data from Stein *et al.* (2013) is used for comparisons. Specifically, datasets are compared in terms of the distribution of alpha-diversities across samples, differential abundance between real and simulated datasets, and sparsity of various samples. Since the realism of MiSDEED’s simulations can be quite sensitive to the quality of the gLV parameter inference described earlier, we perform the comparisons to empirical data side by side with comparisons to gLV parameters inferred from MiSDEED simulations.

## 3 Discussion

MiSDEED dispatches the steady-state of classical microbiome power analysis, instead offering a flexible framework for rapidly generating large amounts of realistic microbial trajectory data which can be used for study design, transfer learning and algorithm development. MiSDEED relies on gLV, which fails to model ecosystems with a high degree of mutualism and therefore limits some of its uses; however, its modular design allows makes it possible to use other models. Future development will focus on expanding code-free interfaces to MiSDEED; more flexible modeling assumptions for broader use cases, including nonuniform time points, individual variation in interaction matrices and growth rates, and population clusters; alternatives to gLV-based modeling for dynamics like mutualism; methods for modeling spatial ecological dynamics; phylogenetically related dimensions on nodes; and investigation into the value of MiSDEED-generated data for transfer learning and algorithm development.

## Funding

This material is based upon work supported by the National Science Foundation Graduate Research Fellowship to P.C. under grant no. DGE-2036197, NIH/NCI Grant No. U54CA209997 Driving Biological Projects, and Columbia University’s 2020/2021 Data Science Institute Seed Grant.

*Conflict of Interest*: none declared.

## Data availability

All data used in this study are available at github.com/pchlenski/misdeed.  
